# The ageing central nervous system in multiple sclerosis: the imaging perspective

**DOI:** 10.1093/brain/awae251

**Published:** 2024-07-24

**Authors:** Massimo Filippi, Paolo Preziosa, Frederik Barkhof, Olga Ciccarelli, Andrea Cossarizza, Nicola De Stefano, Claudio Gasperini, Ruth Geraldes, Cristina Granziera, Lukas Haider, Hans Lassmann, Monica Margoni, Giuseppe Pontillo, Stefan Ropele, Àlex Rovira, Jaume Sastre-Garriga, Tarek A Yousry, Maria A Rocca

**Affiliations:** Neuroimaging Research Unit, Division of Neuroscience, IRCCS San Raffaele Scientific Institute, 20132 Milan, Italy; Neurology Unit, IRCCS San Raffaele Scientific Institute, 20132 Milan, Italy; Neurorehabilitation Unit, IRCCS San Raffaele Scientific Institute, 20132 Milan, Italy; Neurophysiology Service, IRCCS San Raffaele Scientific Institute, 20132 Milan, Italy; Vita-Salute San Raffaele University, 20132 Milan, Italy; Neuroimaging Research Unit, Division of Neuroscience, IRCCS San Raffaele Scientific Institute, 20132 Milan, Italy; Neurology Unit, IRCCS San Raffaele Scientific Institute, 20132 Milan, Italy; Vita-Salute San Raffaele University, 20132 Milan, Italy; Department of Radiology and Nuclear Medicine, Amsterdam UMC, Vrije Universiteit, 1081 HV Amsterdam, The Netherlands; Queen Square Institute of Neurology and Centre for Medical Image Computing, University College London, London WC1N 3BG, UK; Queen Square MS Centre, UCL Institute of Neurology, UCL, London WC1N 3BG, UK; NIHR (National Institute for Health and Care Research) UCLH (University College London Hospitals) BRC (Biomedical Research Centre), London WC1N 3BG, UK; Department of Medical and Surgical Sciences for Children and Adults, University of Modena and Reggio Emilia, 42121 Modena, Italy; Department of Medicine, Surgery and Neuroscience, University of Siena, 53100 Siena, Italy; Department of Neurosciences, S Camillo Forlanini Hospital Rome, 00152 Rome, Italy; Clinical Neurology, John Radcliffe Hospital, Oxford University Foundation Trust, Oxford OX3 9DU, UK; Nuffield Department of Clinical Neurosciences, John Radcliffe Hospital, University of Oxford, Oxford OX3 9DU, UK; Department of Neurology, University Hospital Basel and University of Basel, 4031 Basel, Switzerland; Research Center for Clinical Neuroimmunology and Neuroscience Basel (RC2NB), University Hospital Basel and University of Basel, 4031 Basel, Switzerland; Translational Imaging in Neurology (ThINk) Basel, Department of Biomedical Engineering, University Hospital Basel and University of Basel, 4031 Basel, Switzerland; Queen Square Institute of Neurology and Centre for Medical Image Computing, University College London, London WC1N 3BG, UK; Department of Biomedical Imaging and Image Guided Therapy, Medical University of Vienna, 1090 Vienna, Austria; Center for Brain Research, Medical University of Vienna, 1090 Vienna, Austria; Neuroimaging Research Unit, Division of Neuroscience, IRCCS San Raffaele Scientific Institute, 20132 Milan, Italy; Neurology Unit, IRCCS San Raffaele Scientific Institute, 20132 Milan, Italy; Neurorehabilitation Unit, IRCCS San Raffaele Scientific Institute, 20132 Milan, Italy; Department of Radiology and Nuclear Medicine, Amsterdam UMC, Vrije Universiteit, 1081 HV Amsterdam, The Netherlands; Queen Square Institute of Neurology and Centre for Medical Image Computing, University College London, London WC1N 3BG, UK; Department of Advanced Biomedical Sciences, University “Federico II”, 80138 Naples, Italy; Department of Neurology, Medical University of Graz, 8010 Graz, Austria; Neuroradiology Section, Department of Radiology, Hospital Universitari Vall d'Hebron, 08035 Barcelona, Spain; Neurology Department and Multiple Sclerosis Centre of Catalunya (Cemcat), Vall d'Hebron University Hospital, Universitat Autònoma de Barcelona, 08035 Barcelona, Spain; Lysholm Department of Neuroradiology, UCLH National Hospital for Neurology and Neurosurgery, Neuroradiological Academic Unit, UCL Institute of Neurology, London WC1N 3BG, UK; Neuroimaging Research Unit, Division of Neuroscience, IRCCS San Raffaele Scientific Institute, 20132 Milan, Italy; Neurology Unit, IRCCS San Raffaele Scientific Institute, 20132 Milan, Italy; Vita-Salute San Raffaele University, 20132 Milan, Italy

**Keywords:** multiple sclerosis, ageing, MRI, comorbidities, diagnosis, progression

## Abstract

The interaction between ageing and multiple sclerosis is complex and carries significant implications for patient care. Managing multiple sclerosis effectively requires an understanding of how ageing and multiple sclerosis impact brain structure and function. Ageing inherently induces brain changes, including reduced plasticity, diminished grey matter volume, and ischaemic lesion accumulation. When combined with multiple sclerosis pathology, these age-related alterations may worsen clinical disability. Ageing may also influence the response of multiple sclerosis patients to therapies and/or their side effects, highlighting the importance of adjusted treatment considerations. MRI is highly sensitive to age- and multiple sclerosis-related processes. Accordingly, MRI can provide insights into the relationship between ageing and multiple sclerosis, enabling a better understanding of their pathophysiological interplay and informing treatment selection. This review summarizes current knowledge on the immunopathological and MRI aspects of ageing in the CNS in the context of multiple sclerosis. Starting from immunosenescence, ageing-related pathological mechanisms and specific features like enlarged Virchow-Robin spaces, this review then explores clinical aspects, including late-onset multiple sclerosis, the influence of age on diagnostic criteria, and comorbidity effects on imaging features. The role of MRI in understanding neurodegeneration, iron dynamics and myelin changes influenced by ageing and how MRI can contribute to defining treatment effects in ageing multiple sclerosis patients, are also discussed.

## Teaser

Filippi *et al.* explore the impact of ageing in the context of multiple sclerosis, covering topics such as immunosenescence, diagnostic criteria in older patients, and the effects of comorbidities, as well as the use of MRI to understand neurodegeneration, iron dynamics and myelin changes, and to monitor treatment response.

## Introduction

Multiple sclerosis is an inflammatory, demyelinating and neurodegenerative disease characterized by the progressive accumulation of CNS damage.^1^ On the other hand, as individuals age, their brains tend to show alterations, including limited plasticity, intra- and extracellular protein accumulation, reduced grey matter (GM) volume, increased white matter (WM) abnormalities and ischaemic lesions.^2^ In patients with multiple sclerosis, the interplay between the disease and ageing is complex and has substantial implications as it may determine cumulative and potentiation effects that exacerbate the pathophysiological changes observed in both conditions separately.

By acting in parallel, these two factors may contribute to the overall cumulative burden of CNS pathology. The physiological neurodegenerative phenomena occurring with ageing can be compounded by the inflammatory, demyelinating and neurodegenerative processes of multiple sclerosis, leading to a greater overall impact on brain health. Age-related decline in neuroplasticity and regenerative capacity may exacerbate the neuronal damage and functional impairments caused by multiple sclerosis. This detrimental potentiation effect means that older patients with multiple sclerosis might experience more severe disease progression and disability compared to younger individuals with the same disease duration. Conversely, multiple sclerosis can accelerate ageing-related features. Chronic inflammation, demyelination and neurodegeneration associated with multiple sclerosis may lead to premature brain ageing. This accelerated ageing can manifest as earlier onset of age-related cognitive decline, increased brain atrophy, and other neurodegenerative changes typically seen in older adults.

Understanding the interplay between ageing and multiple sclerosis mechanisms is crucial for effective management of patients. This is particularly relevant because the proportion of patients experiencing a clinical onset of multiple sclerosis at an advanced age has increased in recent years.^3-5^ Furthermore, patients with multiple sclerosis are more likely to reach an older age due to early diagnosis and early initiation of effective treatments, as both ageing and multiple sclerosis affect brain structure and function and their combination may have detrimental additive and even multiplicative effects. Ageing may also influence the management of patients with multiple sclerosis as it is associated with increased risk of treatment side effects and lower occurrence of clinical relapses and new lesions on MRI scans,^6^ thus emphasizing the need for age-adjusted treatment considerations.^7^

MRI is highly sensitive to age- and multiple sclerosis-related processes and it plays a crucial role in tracking disease progression, CNS damage accumulation, and treatment efficacy. Accordingly, MRI can provide insights into the relationship between ageing and multiple sclerosis, enabling a better understanding of the underlying pathophysiological processes and their interplay, and guiding treatment.

An international meeting within the Magnetic Resonance Imaging in Multiple Sclerosis (MAGNIMS) network (https://www.magnims.eu/) was held on the 10 November 2023, which involved neurologists, immunologists, pathologists, physicists and (neuro)radiologists with expertise in multiple sclerosis and MRI (Supplementary material) to summarize the most recent knowledge on the immunopathological and neuroimaging aspects of ageing in the CNS in the context of multiple sclerosis. The key aspects discussed in the meeting included the most recent evidence regarding immunosenescence, ageing-related pathological mechanisms, and specific features like enlarged Virchow-Robin spaces and glymphatic system dysfunction. Clinical aspects, including late-onset multiple sclerosis, the influence of age on diagnostic criteria, and comorbidity effects on imaging features were also reviewed. Finally, the role of MRI in understanding neurodegeneration, iron dynamics and myelin changes influenced by ageing and how MRI can contribute to defining treatment effects in ageing multiple sclerosis patients were examined.

Experts provided a summary related to each topic (see Supplementary Table 1 for search strategy and selection criteria). A group consensus was reached during the meeting and summarized in a first draft, which was circulated among the speakers and additional experts in the field for critical discussion and revision.

## Immunopathology of ageing CNS in multiple sclerosis

### Senescence of the immune system

Ageing is characterized by an irreversible physiological decline in immunological defence that is caused by several immune modifications resulting, among others, in the exacerbation of the severity of chronic diseases.^8,9^ Numerous causal determinants of age-related changes that occur in many cell types at both the molecular and cellular levels have been described, and the characteristics of many of them resemble the immune changes that occur in patients with multiple sclerosis.^9^

Immunological ageing is characterized by phenotypical and functional changes in different cell populations, including myeloid cells as well as T and B lymphocytes, which can assume the so-called senescence-associated secretory phenotype (SASP) that indicates the onset of senescent cells that become able to secrete high levels of pro-inflammatory cytokines and chemokines, along with a variety of molecules able to modulate immune response, including growth factors, proteases, exosomes containing enzymes, microRNA, DNA fragments, among others. In turn, SASP phenotype can maintain a chronic, sterile, low-grade inflammation that develops in the absence of overt infections and has been defined as ‘inflammageing’.^10-12^ This is a systemic phenomenon, the trigger of which has not been yet clarified, but in which both endogenous and exogenous factors, namely genetics, infections and the environment, including diet, play a crucial role. Similarly, in the pathogenesis of multiple sclerosis inherited susceptibility accounts for about one-third of the overall disease risk, while factors such as infections, nutrition, smoking and vitamin D levels can facilitate the onset of the disease in genetically vulnerable persons.

Starting from cells belonging to innate immunity, inflammageing causes and maintains cell activation.

Ageing microglia often exhibit dystrophic morphology, characterized by retracted and less complex processes.^9,13-15^ These changes are thought to impair their surveillance capabilities. Moreover, phagocytic activity of microglia declines with age, reducing their efficiency in clearing cellular debris and damaged cells. Additionally, aged microglia show a dysregulated response to injury and disease, often leading to an exaggerated inflammatory response, with the adoption of a more pro-inflammatory phenotype. Consequently, in patients with multiple sclerosis, the aged CNS environment may promote persistent microglia activation not only in chronic active lesions, but also in the normal-appearing WM.

Ageing is also associated with an increase in the density of CNS-associated macrophages (CAMs), which include meningeal, choroid plexus and perivascular macrophages.^14^ Such changes might impact their roles in maintaining CNS homeostasis and immune surveillance. Similar to microglia, CAMs also tend towards a pro-inflammatory state during ageing and to show reduced efficiency in clearing debris and maintaining the blood–brain barrier.

With ageing, T lymphocytes increasingly display markers related to T helper (Th) 1 and Th17 activity, as well as changes in cytotoxicity and decreased regulatory capability.^11^ Also, inflammageing creates a microenvironment that predisposes to the development of neurodegenerative diseases, with progressive dysfunction and degeneration of neurons in the CNS. Similarly, in multiple sclerosis, the inflammation that is triggered by the first autoimmune reaction in the CNS is capable of causing an imbalance between the autoinflammatory and autoregulatory capabilities of CD4+ and CD8+ T lymphocytes that infiltrate the CNS itself. In turn, they become able to activate microglia, astrocytes and monocytes present in the microenvironment, promoting neuro-inflammation. Of note, this phenomenon seems to be self-limiting, as focal inflammatory lesions become less frequent with the age and the duration of multiple sclerosis, even if demyelinated lesions can remain chronically active. This could suggest that inflammatory processes, i.e. cells of the innate immunity, trigger modifications of the microenvironment that cause irreversible damage to the cells present in that area, whose functional alterations (such as those affecting energy metabolism, mitochondrial functionality, intercellular communications, among others^8^) cause and maintain degenerative processes and the eventual onset of new demyelinated lesions in the absence of strictly inflammatory molecules and cells.

During ageing, thymic involution and stem cell exhaustion lead to complex remodelling of key immune functions that can be identified by measuring the so-called ‘immune risk phenotype’. This includes a CD4:CD8 ratio of <1, poor T-cell proliferative responses, increased number of late differentiated CD8+ cells, low B cell numbers, and cytomegalovirus-seropositivity.^16,17^ These changes reflect the decreased effectiveness in protecting the host from external and internal threatens, such as different types of pathogens, or the accumulation of damage that disturb cellular homeostasis and cause either degeneration at the organelle or cell level, and eventually lead to the onset of autoimmune phenomena. Such phenomena can be controlled, at least in part, by regulatory T lymphocytes (Tregs, both CD4+ and CD8+), whose role in physiological ageing is still controversial,^17,18^ but which have a fundamental role in counteracting autoimmunity and maintaining tolerance but display decreased functionality during inflammageing. In multiple sclerosis, the number of these cells seems to remain unchanged, whereas their functional suppressive capabilities are decreased and their tendency to produce Th1-type inflammatory molecules is increased.^19,20^ As a result, autoimmune clones and the phenomena that follow the initial damage and lead to neurodegeneration are no longer controllable.

Concerning B cells, besides becoming plasma cells that produce antibodies, they exert other critical regulatory functions in activating or suppressing immune responses. With age, they can secrete inflammatory molecules such as tumour necrosis factor (TNF) and interleukin 6 (IL-6), produce autoantibodies (i.e. anti-DNA, not necessarily correlated to an autoimmune disease) and expand clones after chronic viral infections such as those by Epstein–Barr virus (EBV) or cytomegalovirus (CMV).^21^ In the pathogenesis of multiple sclerosis, such cells play a pivotal role and indeed several studies have demonstrated the presence of self-reacting, immunoglobulin-producing B cell clones in the CSF, meninges and brain. Thus, anti-CD20 therapies, which spare plasma cells but deplete B lymphocytes, are indeed extremely effective in treating multiple sclerosis and, interestingly, the immunosuppressive cytokine IL-10 produced by plasma cells has a protective value when present in multiple sclerosis lesions.

Finally, in the non-coding part of the genome of human senescent cells, the most recently integrated endogenous retroviruses (ERVs), i.e. HERVK (HML-2), are unlocked to transcribe viral genes and produce retrovirus-like particles (RVLPs), which become a message to elicit senescence phenotypes in young cells. The activation of ERVs was recently described in tissues and serum from aged donors, and indeed the repression of ERV activity ameliorates cellular senescence and degeneration of different tissues and, in turn, ageing of the individuals,^22^ likely opening a new chapter in the search of strategies to improve immune performances during ageing.

### Pathological mechanisms and ageing in multiple sclerosis

Improvements in general healthcare and multiple sclerosis treatment have increased life expectancy of patients with multiple sclerosis during the last decades. In a Norwegian study including 1388 multiple sclerosis patients with onset from 1953 to 2012, the standardized mortality ratios (SMR) of multiple sclerosis relative to the general populations dropped gradually from 3.1 for disease onset during 1953–74, to 2.6 for disease onset during 1975–96 and 0.7 for disease onset during 1997–2012.^23^ Similarly, in a Danish study including 18 847 patients with definite or probable multiple sclerosis and onset from 1950 to 1999, the SMR of multiple sclerosis relative to the general populations dropped gradually from 4.48 in the 1950–59 onset cohort to 1.80 in the 1990–99 onset cohort.^24^ Moreover, mean age of death gradually increased from 50.6 years in patients that died between 1950 and 1959 to 65.4 years in those that died between 2000 and 2009.^24^ This has also been confirmed by a recent systematic analysis for the Global Burden of Disease Study, which showed an 11.5% global decrease in age-standardized death rates in 2016 compared to 1990.^25^

This implies that most patients reach an age at which age-related health problems may interfere with the disease process. This interference may happen coincidentally or through the direct interaction of disease-specific mechanisms and ageing-related brain damage.

Multiple sclerosis is a chronic inflammatory disease of the CNS leading to demyelination and neurodegeneration. Inflammation is dominated by CD8^+^ T cells and B-cell infiltrates, entering the CNS in active lesions but residing within the brain and spinal cord as tissue resident memory cells associated with progressive tissue damage.^26,27^ Demyelination and neurodegeneration are induced by a cascade of microglia activation, oxidative injury and mitochondrial dysfunction, resulting in a state of metabolic energy deficiency.^28^

There are no qualitative differences in the multiple sclerosis pathology between different forms or stages of the disease. Thus, the entire spectrum of multiple sclerosis typical alterations can be seen in the brain and spinal cord of patients who died during the relapsing or the progressive stage. However, systematic studies, based on a large patient cohort and lesion sample, revealed major quantitative differences.^29,30^ Active lesions with massive macrophage infiltration are mainly seen in the early disease stages but are rare in patients with progressive disease. Chronic active lesions and, more specifically, the slowly expanding lesions slowly increase with disease duration and peak at the transition stage between relapsing and progressive disease, while the extent of remyelination remains similar throughout all disease stages. A gradual increase in incidence with a peak in the progressive stage of the disease is also seen for cortical lesions and diffuse injury in the normal appearing WM.^31^

In the early stages of the disease, new multiple sclerosis lesions can arise at any sites in the brain and spinal cord, but with disease progression they tend to accumulate in the periventricular WM and subpial layers of the cortex,^32^ and lateral or posterior columns of the spinal cord. Pathological changes associated with disease progression consist of gradual chronic expansion for years of pre-existing lesions^29,30,33^ in WM and GM, and slow accumulation of diffuse inflammation and neurodegeneration in the normal appearing WM or GM.

Recent genetic studies have identified four potential candidate genes associated with disease severity in multiple sclerosis,^34^ which may also play a role in disease progression. Zink finger protein 386 mediates transcriptional repression of unintegrated viral DNA (possibly EBV and HERV-W), dysferlin and dynamin 3 are involved in the repair of cell membrane damage, whereas phosphatidylinositol glycan anchor (GPI) biosynthesis class C protein is important for the expression of GPI anchored membrane proteins. Thus, the latter three may be involved in the repair of damaged cells or cell processes.^35^

Progressive brain damage in multiple sclerosis can be augmented by mechanisms related to ageing, disease duration or the accumulation of brain damage. Oxidative injury and mitochondrial dysfunction also propagate brain damage in ageing and in age-related vasculo-ischaemic diseases,^36^ and this is further amplified by age-related accumulation of iron within the human brain.^37,38^ Similarly, microglia activation is prominent in age-related neurodegeneration^39^ and susceptibility to neurodegenerative diseases, such as Alzheimer's disease, is in part associated with polymorphisms in genes linked to microglia function.^40,41^ Chronic brain inflammation may induce misfolded proteins in neurons, which may contribute to neurodegeneration.^42^ Finally, remyelination capacity decreases with ageing and chronic brain inflammation.^43,44^

Thus, comorbidities with vascular and neurodegenerative diseases are likely to enhance clinical disease and neurodegeneration in ageing multiple sclerosis patients. As mentioned above, this is particularly relevant for vasculo-ischaemic diseases,^45^ which share molecular mechanisms with disease progression in multiple sclerosis. In contrast to experimental studies, which suggest that demyelination propagates amyloid deposition^46^ and that amyloid-β oligomers are toxic for myelin,^47^ no significant difference was noted in the development and phenotype of Alzheimer's disease associated neuropathology between patients with long-lasting progressive multiple sclerosis and age-matched controls.^48^ However, the data also document the occurrence of Alzheimer's disease in ageing multiple sclerosis patients and this emerging co-pathology may amplify cognitive disabilities.

### Enlarged Virchow-Robin spaces and glymphatic impairment

**Figure 1:**
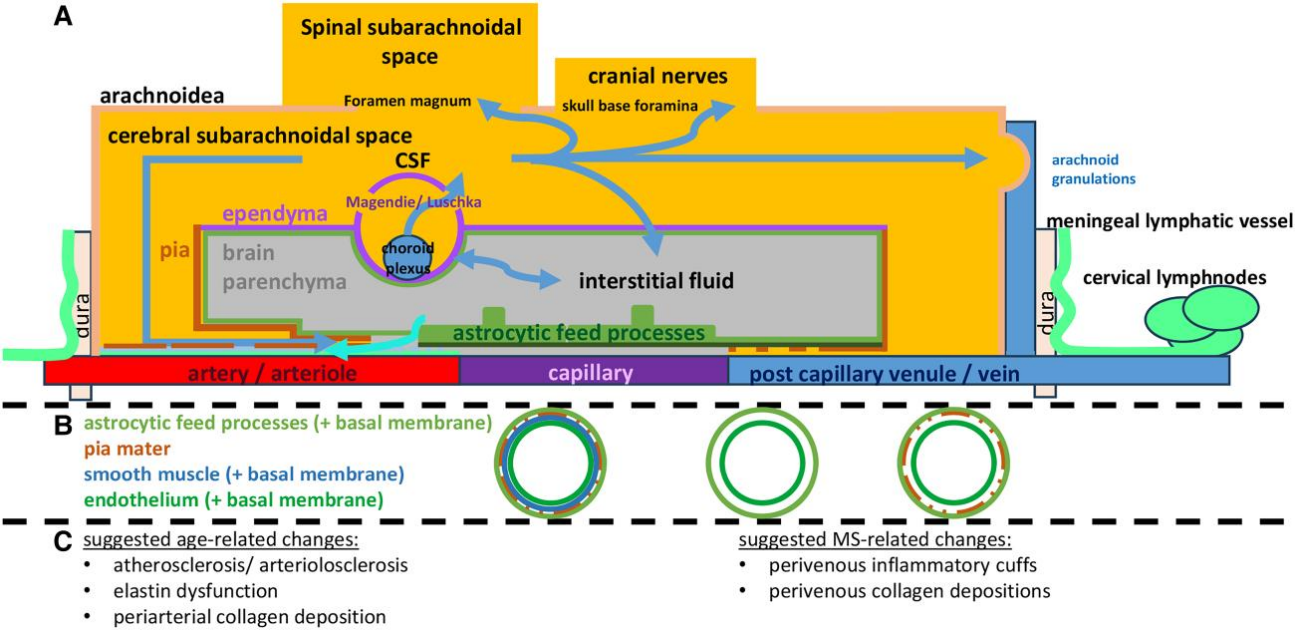
**The ageing perivascular compartment in multiple sclerosis.** Perivascular spaces (PVS), which are implicated in brain waste removal, are involved in ageing and multiple sclerosis (MS) at different levels. CSF, produced in the choroid plexus, exchanges with brain interstitial fluid. In addition to the established CSF exit pathways along the spinal subarachnoid space, cranial nerves and arachnoid granulations, a portion of CSF flows into the brain parenchyma via the periarterial space. This flow is part of the glymphatic drainage pathway, illustrated along the arteries and through pial fenestrations. Concurrently, protein degradation products are conveyed within the muscularis of arteries, moving counter to the direction of blood flow, into the subarachnoid arteries. This process is part of the intramural peri-arterial drainage pathway, represented in blue along the artery (**A**). At the arterial and arteriolar level, cross-sectional views reveal that the perivascular space comprises the astrocytic end-feet processes (including their corresponding basement membrane), the pia mater (which becomes increasingly fenestrated closer to the capillary level), smooth muscle cells and the endothelium (each with their respective basement membranes). Within capillaries, the perivascular space is defined by the shared basement membranes of the astrocytic end-feet processes and the endothelium. The CSF-filled subarachnoid spaces are also evident along veins and venules, where the layers of smooth muscle cells are largely absent (**B**). Age-related factors, such as atherosclerosis/arteriolosclerosis, elastin dysfunction and periarterial collagen deposition, have been implicated in vascular stiffness, diminished debris transport capacity, and an increased barrier to oxygen delivery. In multiple sclerosis, perivascular changes include collagen deposition and perivenous inflammatory infiltrates that come into contact with CSF (**C**).

Perivascular or Virchow-Robin spaces are fluid, or extracellular matrix filled spaces (areas) between the basement membranes of the astrocytic feed processes and the brain endothelium of arteries, capillaries and veins of the CNS (Fig. 1).^49^

Perivascular spaces are involved in brain waste clearance processes, by allowing CSF entry from the subarachnoid space into the peri-arterial compartment. This process is facilitated by aquaporin 4 (AQP4)-dependent fluid transfer to the brain interstitial fluid. Additionally, an intramural peri-arterial drainage pathway has been suggested, transporting debris from the interstitium against the arterial blood flow direction, into the smooth musculature of subarachnoidal arteries (Fig. 1).^50^

Under normal conditions, perivascular spaces in the deep WM are not visualized on brain MRI scans using standard clinical protocols at 1.5 and 3 T. However, enlarged perivascular spaces (ePVS) become more prevalent with age and are associated with a broad range of neurological conditions.^51^ Different mechanisms for perivascular space enlargement have been suggested in the context of multiple sclerosis, including perivenous inflammation, brain atrophy, expansion of perivascular extracellular matrix and features of brain ageing, such as cerebral small vessel disease (cSVD), including debris accumulation and arterial tortuosity.^52^

Perivenous inflammation is a key feature of multiple sclerosis lesions and in histopathological sections, perivenous inflammatory infiltrates in lesions can reach counts of >200 cells on an axial section [mean = 40.9, standard deviation (SD) = 36.7],^53^ suggesting that this feature could be visualized with MRI. Furthermore, systemic inflammation has repeatedly been associated with perivascular space enlargement across several neurological conditions^54^ and multiple sclerosis cohorts. Among individuals with high disease activity, correlations with gadolinium-enhancing lesions have been reported.^55^ By contrast, peri-arteriolar extracellular matrix depositions and cSVD features in ePVS have been identified in multiple sclerosis,^56^ though without histological validation in active multiple sclerosis.

The decrease of CSF clearance^57^ and reduction in diffusivity along perivascular spaces in multiple sclerosis have been shown to be pronounced within the first 4 years, correlating with higher WM lesion volume, brain volume loss and worse disability.^58^ Reduced clearance of CSF-derived toxic molecules may lead to gradients of tissue injury along CSF surfaces.^59^

cSVD is known to correlate with age^60^ and is increased in multiple sclerosis.^45^ cSVD-related WM lesions are associated with, and grow around, ePVS in both normal ageing^61^ and multiple sclerosis.^56^ The decreasing diagnostic accuracy of the ‘central vein sign’ (CVS) with age and presence of ePVS^62^ highlight the limited specificity of MRI for WM lesions in older multiple sclerosis patients, likely hindering our understanding of age- and cSVD-related brain involvement in multiple sclerosis, its progression and therefore the applicability of diagnostic criteria.

Overall, while data on the contribution of vascular ageing to tissue damage in multiple sclerosis remain limited, there is evidence supporting the hypothesis of an initial inflammation associated with (potentially perivenous) perivascular space enlargement. This is followed by depositions of extracellular matrix components in the perivascular space, decreased perivascular diffusivity in early disease stages and accelerated periarteriolar cSVD, associated with brain atrophy and global WM lesion burden.

## Clinical aspects of ageing CNS in multiple sclerosis

### Late-onset multiple sclerosis

While multiple sclerosis is typically diagnosed in young adulthood, recent epidemiological studies have revealed that 5–20% of patients experience their first symptom at older ages.^4,5,63,64^ This condition is commonly referred as late-onset multiple sclerosis (Table 1).^4,63,64^ At present, there is no unified consensus on the cut-off of age at onset for defining late-onset multiple sclerosis,^64^ however, the majority of authors consider it as late-onset multiple sclerosis forms of the disease with a clinical presentation after the age of 50.^4,63-68^

**Table 1:** Clinical and pathological features of late-onset multiple sclerosis

Definition	No unified consensus on the cut-off of age at onset
	Generally considered as those cases with disease onset after 50 years of age
Possible underlying pathophysiological mechanisms	More limited overt inflammatory activity
	More severe neurodegenerative phenonema (e.g. neuro-axonal loss)
	Less efficient remyelination capacity
	More limited CNS reserve and neuroplasticity
Symptoms at clinical presentation	High frequency of spinal cord involvement
	High proportion of progressive forms
Disease course	More severe disease course and faster disability progression
	Significantly shorter time to reach clinically-relevant milestones of disability
	Lower prevalence of clinical relapses and new white matter lesions
Cognitive impairment	Impairment in visual learning and memory
Comorbidities	High incidence of diabetes, hypertension and hyperlipidaemia
	High prevalence of depression

Several studies have attempted to delineate the most common clinical features at initial presentation, disease course and progression of these patients.^65,67,69,70^ Compared to adult-onset multiple sclerosis, late-onset multiple sclerosis is commonly associated with a more severe disease course and faster disability progression,^71^ with a significantly shorter time to reach clinically relevant milestones of disability,^72,73^ a higher proportion of progressive disease clinical phenotypes^65,70^ and lower frequency of inflammatory relapses.^67^ Several factors may contribute to explain these differences. First, in patients with late-onset multiple sclerosis, the involvement of the spinal cord at clinical onset is typically more frequent than in younger age classes, partially explaining the worse outcome.^5,71^ Second, young multiple sclerosis patients exhibit some capability to compensate for pathological changes during the early inflammatory stages, such as through remyelination. However, in the ageing multiple sclerosis brain, compensatory reserve declines, ultimately resulting in a faster disease progression in elderly multiple sclerosis.^4^

A recent work that explored the histopathological differences in multiple sclerosis patients by age of onset revealed that patients with late-onset multiple sclerosis had fewer actively demyelinating WM lesions (including both active or chronic active) and less leptomeningeal and perivascular inflammation compared to adult-onset multiple sclerosis patients.^74^ However, both groups had a similar volume of cortical lesions, which represented a greater proportion of the total lesion volume in patients with late-onset multiple sclerosis.^74^ Neuron density was also similar in both groups except in the cingulate gyrus and the thalamus, where patients with late-onset multiple sclerosis had significantly lower density.^74^ Differently from patients with adult-onset multiple sclerosis, no significant association between thalamic neuron density and demyelination or inflammation was found in patients with late-onset multiple sclerosis. Moreover, an older onset was characterized by an already reduced neuron density in the pons and thalamus. These findings suggest that a later onset of the disease may be preceded by a prolonged prodromal phase with lower inflammatory demyelinating activity compared to adult-onset multiple sclerosis, culminating in a more neurodegenerative form of the disease at breakthrough.^74^

Additionally, ‘inflammageing’ may contribute to brain tissue damage, promoting the accumulation of clinical disability.^10^ Indeed, recent findings suggest that microglia already assume an activated state during biological ageing,^75^ thus possibly promoting a receptive setting for the development of pathogenic microglia following multiple sclerosis onset. This chronically inflamed environment could be poorly conducive to remyelination and could contribute to a more rapid development of irreversible disability.^9^

Finally, as in the general population, ageing in multiple sclerosis patients is accompanied by the development and accumulation of comorbidities. Rising incidence of diabetes, hypertension and hyperlipidaemia has been described in multiple sclerosis patients, with an upward trend associated with advancing age.^76^ These comorbidities interact with multiple sclerosis pathology, potentially complicating disease diagnosis, treatment management and prognosis, as discussed later.^4^

Regarding the cognitive profile of patients with late-onset multiple sclerosis, some studies have demonstrated a comparable frequency and pattern of cognitive deficits between this group and patients with adult-onset multiple sclerosis.^67^ On the contrary, other studies have shown more pronounced cognitive deficits in late-onset multiple sclerosis compared to younger patients.^77^ These deficits include impairment in visual learning and memory domains,^53^ and a higher prevalence of depressive symptoms.^65^ These differences may be attributed to the presence of comorbidities and age-related neurodegeneration.^78,79^ One study found severe cortical, cerebellar and brainstem atrophy in patients with late-onset multiple sclerosis with cognitive impairment.^77^

Taken together, the clinical and cognitive profiles of patients with late-onset multiple sclerosis suggest a form of the disease that is characterized by pronounced neurodegenerative processes and a high degree of cognitive impairment. These considerations suggest that diagnosis, monitoring and treatment of late-onset multiple sclerosis present unique challenges.

### Multiple sclerosis diagnostic criteria in aged patients

The current diagnostic criteria for multiple sclerosis, i.e. the 2017 revision of the McDonald criteria,^80^ have been validated primarily using data from adult patients under 50 years of age with a typical clinically isolated syndrome (CIS) suggestive of multiple sclerosis and no comorbidities. However, healthy individuals older than 50 years often exhibit incidental T_2_-hyperintense WM lesions in the brain, possibly due to age-related comorbidities.^81,82^ These lesions may be indistinguishable from multiple sclerosis demyelinating lesions, they may substantially contribute to the overall WM lesion burden in multiple sclerosis patients, and they may be included in the count required to define the fulfilment of dissemination in space (DIS) criteria.^80^

**Figure 2:**
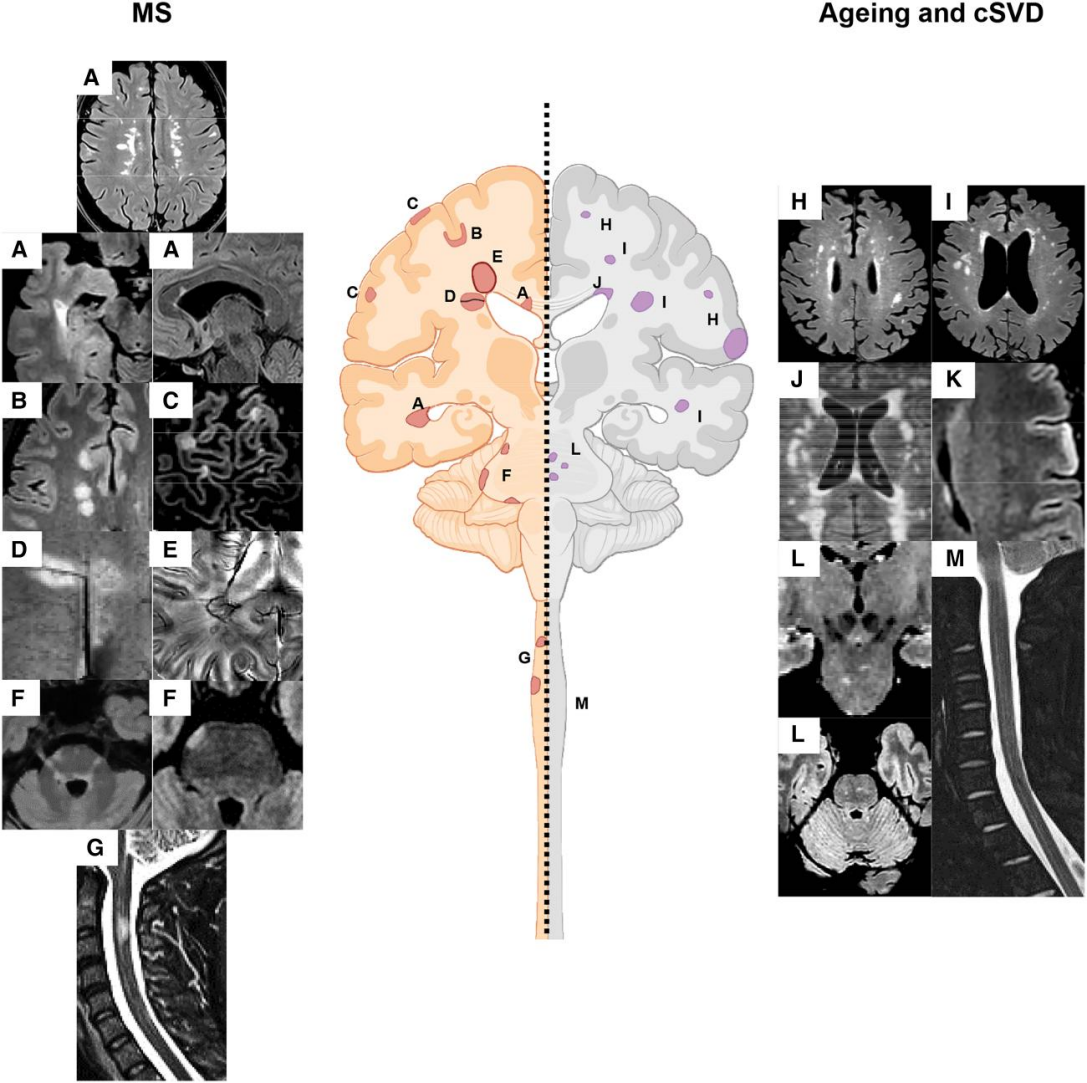
**Summary of the typical lesional MRI findings in multiple sclerosis compared to ageing and cerebral small vessel disease.** Typical multiple sclerosis (MS) lesions include (**A**) periventricular lesions, (**B**) juxtacortical and cortical lesions, (**C**) white matter (WM) lesions showing the central vein sign (CVS), (**E**) paramagnetic rim lesion (PRLs), (**F**) infratentorial lesions mainly located at the periphery, close to the CSF, and (**G**) spinal cord lesions. Typical lesions occurring with ageing and cerebral small vessel disease (cSVD) include (**H**) subcortical WM lesions, (**I**) deep WM lesions, (**J**) periventricular lesions and ‘capping’, (**K**) cortical microinfarcts, (**L**) central pontine lesions and (**L**) no spinal cord lesions. See text for further details.

Periventricular lesions and ‘capping’ increase with age, especially in subjects with cSVD (Fig. 2).^83,84^ The requirement for three instead of one periventricular lesions needed to demonstrate periventricular involvement improved the specificity, reduced sensitivity, but had a marginal impact on accuracy of the 2017 McDonald criteria for DIS in CIS patients older than 40–45 years.^35,85^ As a consequence, looking for more than one periventricular lesion may be prudent in older patients with multiple sclerosis, certainly those with cerebrovascular risk factors.^80,86^

Lesions close to the cortex increase with ageing,^81^ but the impact of age on fulfilling the criterion for cortical/juxtacortical involvement for DIS has not yet been explored. However, lesions associated with cSVD usually spare the cortex and juxtacortical U-fibres (Fig. 2) as these regions receive dual blood supply, superficially from cortical penetrating arteries as well as from deeper vessels that ascend from medullary arteries. Therefore, a meticulous assessment of juxtacortical/cortical lesions is crucial for distinguishing multiple sclerosis from other comorbidities, especially in older patients.

Pontine lesions can occur with ageing but they are typically located in the central portions of the pons and medial lemniscus, a distribution characteristic for cSVD (Fig. 2)^82,87,88^ as these regions correspond to vascular border zones, supplied by different penetrating arteries arising from the basilar and superior cerebellar arteries.^89^ Conversely, peripheral pontine lesions are more specific for multiple sclerosis.^82^ Therefore, in older multiple sclerosis patients, especially with cerebrovascular risk factor, peripheral pontine involvement and lesions abutting the fourth ventricle may be useful to discriminate multiple sclerosis-related lesions from those due to other comorbidities (Fig. 2).

Spinal cord lesions are not observed with normal ageing or with age-related comorbidities.^90-92^ Moreover, even though spinal cord arteriolosclerosis has been observed and may contribute to spinal WM pallor and myelin abnormalities, focal microinfarcts and cerebral amyloid angiopathy were not observed within the spinal cord parenchyma.^93^ Consequently, evaluating spinal cord involvement is crucial, especially in older multiple sclerosis patients, for both diagnostic and prognostic purposes.

Among potential diagnostic MRI markers under investigation, a proportion of WM lesions with the CVS (between 35% and 50%) on susceptibility-based imaging or having at least three or six CVS-positive lesions (3- or 6-lesion rule) may help distinguish multiple sclerosis from other conditions (Fig. 2).^94-99^ However, a significantly lower proportion of CVS-positive WM lesions occurs with ageing, with older multiple sclerosis patients (i.e. ≥50 years) having a significantly lower percentage of CVS-positive lesions compared to younger multiple sclerosis patients (61.5% versus 77.5%).^62^ Despite this, age had a minimal effect on fulfilling the different aforementioned CVS criteria, as most multiple sclerosis patients satisfied the different criteria.^62^

Paramagnetic rim lesions (PRLs) (Fig. 2), i.e. lesions showing a paramagnetic rim on susceptibility-based images, are specific to multiple sclerosis, can differentiate multiple sclerosis from other neurological conditions and may predict conversion from CIS to multiple sclerosis.^100^ A recent meta-analysis estimated that the pooled prevalence of PRLs at lesion level was 9.8%, but this showed a significant decrease with advancing age. However, at the patient level, the pooled prevalence of PRLs was 40.6%, and this prevalence was not influenced by age.^101^ Accordingly, although the total number of PRLs decreases with age, the proportion of multiple sclerosis patients with at least one PRL seems stable throughout the lifespan, thus limiting the impact of ageing on this candidate diagnostic marker.

### Comorbidities: effects on imaging features

There are several reasons why the effect of vascular comorbidities on the ageing multiple sclerosis population needs to be considered. First, vascular comorbidities, such as hypertension and hyperlipidaemia, are often present at multiple sclerosis onset but become even more frequent 5 years after multiple sclerosis diagnosis.^102^ These comorbidities increase with age (i.e. hypertension occurs in >50% of people with multiple sclerosis over the age of 60 years) and are associated with brain atrophy, WM lesions and cognitive changes, even in people without multiple sclerosis.^103^ The interaction between comorbidities and multiple sclerosis may explain variability in clinical outcomes; for instance, people with multiple sclerosis who have vascular comorbidities might need a walking aid sooner and may take less time for treatment escalation than those without these comorbidities.^104^ Dual pathology or potentiation of multiple sclerosis-related damage may explain these negative outcomes. In fact, systemic vascular disease showed a stronger association with cSVD in people with multiple sclerosis compared with those without, and the burden of cSVD linked with multiple sclerosis inflammatory activity.^45^ Vascular damage may lead to neuronal loss, as suggested by studies showing that permanent T_1_-hypointense lesions tend to occur in areas of low cerebral perfusion.^105^ In addition, treatments for vascular comorbidities may affect multiple sclerosis imaging outcomes (i.e. people with multiple sclerosis on anti-diabetic drugs showed lower T_2_-hyperintense lesion volume than those not on these treatments).^106^

There have been several cross-sectional and longitudinal studies looking at the effect of vascular comorbidities on MRI outcomes (Table 2).^107,108^ Most studies are small (mainly on CIS or relapsing-remitting multiple sclerosis), with heterogeneous definitions of comorbidity, and often not considering comorbidity treatments or smoking status. Overall, combined vascular scores are associated with a faster brain parenchymal fraction loss. A similar effect was seen for hypertension, ischaemic heart disease and diabetes.^107,108^ In secondary progressive multiple sclerosis, vascular comorbidities are associated with a decrease in normalized whole brain volume.^109^ Discrepant effects of vascular comorbidities on global T_2_-hyperintense lesion volume and contrast-enhancing lesions have been reported^107^ and vascular comorbidities do not appear to affect conversion from CIS into clinically definite multiple sclerosis in young patients.^110^ In face of a new T_2_-hyperintense WM lesion in a multiple sclerosis patient with vascular comorbidities, one could scrutinize its shape and topography. Each vascular comorbidity may affect T_2_ ‘multiple sclerosis-like lesions’, such as Dawson fingers, juxtacortical lesions^81^ or lesions with CVS^62^ differently (i.e. dyslipidaemia is associated with a higher proportion of juxtacortical lesions and hypertension is associated with a lower proportion CVS-positive WM lesions). Vascular comorbidities do not associate with lesions in the peripheral pons, typically affected in multiple sclerosis, but may increase the likelihood of lesions occurring in topographies usually affected by cSVD (i.e. central pons).^82^

**Table 2:** Summary of the effects of vascular comorbidities on MRI outcomes

Comorbidity	WM lesions	Gd-enhancing lesions	Brain volume	Reference(s)
Hypertension	+/−	?	+ (lower BPF, GM and cortical GM volume loss, lateral ventricle enlargement)	Geraldes *et al*.,^81^ Pichler *et al*.,^110^ Jakimovski *et al*.,^111^ Kappus *et al*.,^112^ Lorefice *et al.*^113^
Hyperlipidaemia	+	+/−	+/−	Lorefice *et al*.,^113^ Fitzgerald *et al*.,^114^ Weinstock-Guttman *et al.*^115^
Diabetes	−	?	+ (lower BPF, GM volume, cortical GM volume)	Salter *et al*.,^108^ Lorefice *et al*.,^113^ Fitzgerald *et al.*^114^
Ischaemic heart disease	?	?	+ (GM and cortical GM volume loss)	Kappus *et al.*^112^
Obesity	+/− (T_1_-hypointense lesion volume +, not T_2_-hyperintense lesion volume)	−	+/−	Fitzgerald *et al*.,^114^ Manuel Escobar *et al*.,^116^ Ben-Zacharia *et al*.,^117^ Galioto *et al.*^118^
Grouped vascular comorbidity	+	?	+ (Higher Framingham risk scores—reduced BPF loss over time)	Marrie *et al.*^119^
Count of comorbid conditions	+	?	+/−	Pichler *et al*.,^110^ Fitzgerald *et al.*^114^

+ = the presence of the vascular risk factor (VRF)/VRF score was reported to influence the imaging outcome; +/− = some studies reported that the presence of the VRF/VRF score influences the imaging outcome but not others; − = no association was reported between the presence of the VRF/VRF score and the imaging outcome; ? = insufficient evidence; BPF = brain parenchymal fraction; Gd = gadolinium; GM = grey matter; WM = white matter.

## MRI to investigate pathophysiology in ageing multiple sclerosis patients

### Ageing and brain atrophy in multiple sclerosis

**Figure 3:**
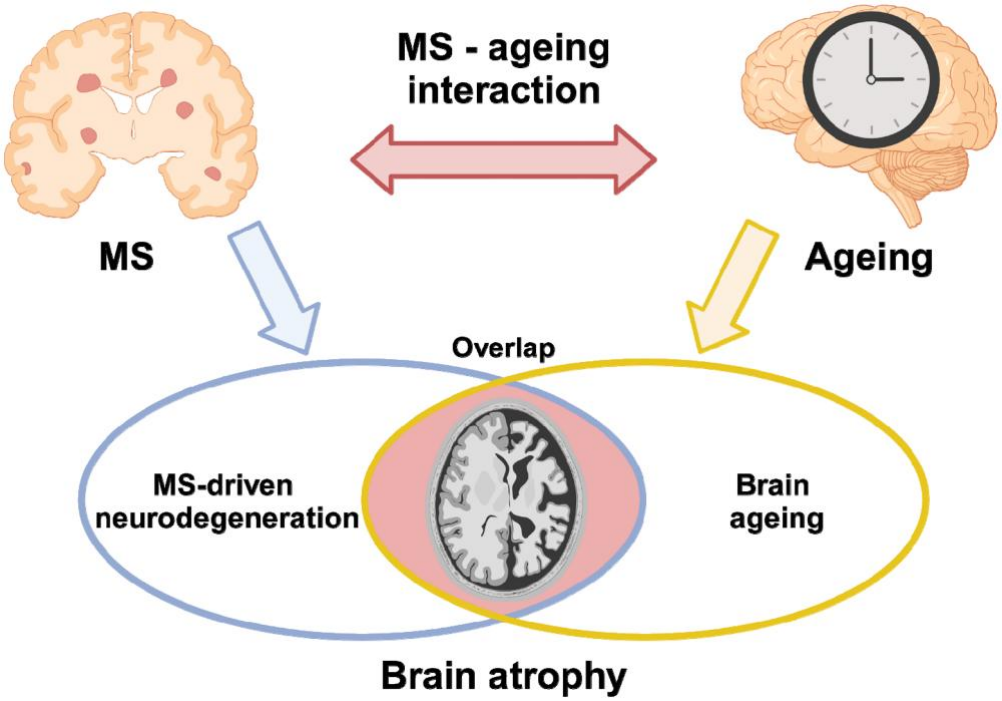
**Schematic representation of the interplay between the effects of multiple sclerosis-related neurodegeneration and ageing on brain atrophy.** Both ageing and multiple sclerosis (MS) are associated with brain atrophy, with partially overlapping patterns (blue arrow). Rather than being simply additive, the effects of ageing and multiple sclerosis on brain atrophy are linked by a complex interaction (red arrow): the relationship between age and brain volume is influenced by multiple sclerosis and encodes disease-related information; ageing shapes multiple sclerosis-related brain atrophy by modifying the disease course and the response to treatment. Created with http://www.biorender.com/.

While age is often treated as a mere confounding variable in neuroimaging-based brain volumetric analyses, the effects of ageing and multiple sclerosis on brain atrophy are closely entangled (Fig. 3). The relationship between age and brain volume is influenced by the disease and encodes disease-related information. Conversely, age is a fundamental modifier of multiple sclerosis clinical course and correlates with the outcomes that define treatment response.^9^ Understanding the complex interaction between brain ageing and neurodegeneration and disentangling their overlapping imaging patterns and underlying mechanisms are the topics of increasing research interest.

Normal ageing-related brain volume loss appears around the age of 30, with rates of ∼0.2% per year, and accelerates after the age of 50–60 up to 0.5% per year (5% per decade).^120^ Against this background, multiple sclerosis is associated with disease-specific volume loss (i.e. atrophy in excess of normal ageing), which starts very early in the disease course, tends to follow specific spatial-temporal patterns, and is linked to poor clinical outcomes.^121,122^ Divergence from normal brain charts is observable as early as the preclinical phase, especially for the thalamus, with normal and multiple sclerosis lifespan trajectories of brain volume change tending to align in the elderly.^123^ Indeed, the proportion of brain atrophy that is attributable to ageing increases over time, while that attributable to multiple sclerosis pathology might decrease with age.^124^ Interestingly, a connection between ageing and multiple sclerosis-related brain atrophy has been demonstrated beyond the purely chronological level: shorter leucocyte telomere length, considered a marker of biological senescence, is associated with brain atrophy independent of chronological age and disease duration, suggesting that biological ageing may contribute to neurological injury in multiple sclerosis.^125^

By flipping the classical paradigm of normative modelling, individual deviations from normal ageing trajectories can also be measured as the difference between neuroimaging-based age predictions relying on machine-learning techniques and chronological age.^126^ Using the brain-age paradigm, various studies have consistently demonstrated that the brains of patients with multiple sclerosis tend to look older than healthy controls on MRI, revealing premature/accelerated ageing.^127,128^ The brain-predicted age difference, proposed as an age-adjusted global measure of brain health, emerged as a promising biomarker in multiple sclerosis, and it correlates with disability scores both cross-sectionally and longitudinally.^127^ However, while the brain-age paradigm offers a window into brain ageing in multiple sclerosis, it may miss disease-specific effects.^127^

In summary, the interaction between disease-specific and age-related brain volume changes remains complex and not completely understood, representing a crucial area for future research.

Moreover, brain age is currently derived globally for the entire brain. In the future, determining brain age for each individual brain parcel could be useful, as brain atrophy associated with multiple sclerosis is non-random and it affects some regions more than others.

### Quantification of iron abnormalities

Iron accumulation in the CNS occurs during physiological ageing as well as in neuroinflammatory and neurodegenerative disorders like multiple sclerosis.^129^ When ferrous iron (Fe^2+^)-content increases in the CNS—originating for example, from micro-haemorrhages or degeneration of oligodendrocytes and myelin—reactive oxygen species (ROS) are produced that provoke metabolic dysfunction, oxidative stress and glutamate Ca^2+^ excitotoxicity.^130^ Therefore, quantifying iron presence is fundamental to assess the extent of neurodegeneration that occurs in ageing and multiple sclerosis.

MRI exploits ‘magnetic susceptibility (χ)’ to assess the presence of iron in the CNS as this metal has the property to strongly increase the local magnetic field. Magnetic susceptibility can be acquired using gradient-echo (GRE) or echo-planar imaging (EPI) sequences, which provide images that can be reconstructed using T_2_* mapping (when multi-echo data are available), susceptibility-weighted imaging (SWI) or quantitative susceptibility mapping (QSM).

Applying QSM, it was possible to understand that iron specifically accumulates in some brain regions during the ageing process.^131^ According to the majority of QSM studies, there is an important iron increase in the putamen with less evidence available for the caudate, substantia nigra and other deep WM nuclei. In the cortex, most studies point to iron accumulation that is especially evident in the frontal-parietal cortex,^131^ with one study showing that layer five in the motor cortex has a particular vulnerability to age-related QSM/iron increase.^132^

It is also important to consider that different iron-sensitive quantitative MRI measures (i.e. quantitative T_2_, T_2_, T_2_* and maps derived from T_2_* data such as QSM) show peaks at different ages.^133^ This points to the need to carefully interpret imaging studies using measures that are sensitive to iron accumulation in the CNS.

In multiple sclerosis patients, iron is stored in oligodendrocytes and myelin in the normal appearing WM and GM, whereas it is also found in microglia/macrophages and astrocytes in active and chronic active lesions.^37^ In contrast to healthy controls, iron appears to decrease with age in the subcortical WM of people with multiple sclerosis,^37^ although it is relatively increased in the peri-plaque tissue.^37^ Similarly, iron transport (Hephaestin) and oxidation (Ceruloplasmin) are increased in surrounding multiple sclerosis lesions.^37^

Interestingly, iron in the basal ganglia appears to increase more over time in CIS versus multiple sclerosis patients (as measured with T_2_* data)^134^ and people with progressive multiple sclerosis exhibit more iron in the basal ganglia than people with relapsing-remitting multiple sclerosis.^135^ However, the thalamus shows a peculiar behaviour with progressive iron decrease—which is more pronounced in secondary progressive multiple sclerosis versus relapsing-remitting multiple sclerosis^136^—even after correction for atrophy.^137^ Last, as previous mentioned, PRLs—a special lesion subtype that shows an iron accumulation at the edge—appear to decrease with age and disease duration.^101^

### Quantification of myelin damage and repair

Assessing myelin damage and repair *in vivo* with MRI has been an ambitious goal for decades. The composition and architecture of myelin and its corresponding electromagnetic properties open the door for several quantitative magnetic resonance techniques. This includes relaxation time mapping, myelin water fraction (MWF) mapping, magnetization transfer (MT) imaging, inhomogeneous MT, and the assessment of molecular diffusion.^138^ Latest developments include higher order diffusion models^139^ and magnetic susceptibility source separation, which is based on the diamagnetic properties of myelin.^140^ Not all of the proposed methods are readily available for clinical application because of long acquisition times, extensive postprocessing requirements or limited sequence availability. Nevertheless, their validation is a fundamental prerequisite before being used as a specific magnetic resonance biomarker for myelin. When considering all post-mortem validation studies carried out to date, the best evidence regarding sensitivity and specificity is given for MWF and MT ratio (MTR), in particular when both the number of tissue samples included in these studies and the correlation factor are taken into account.^141^ However, care should be taken when extrapolating results from validation studies without considering fixation effects, measurement temperature and magnetic field strengths.

Relevant insights into magnetic resonance measures for myelin do not only come from validation studies but also from observations in longitudinal clinical and pre-clinical studies. Several studies have used MTR to track lesion evolution over time in multiple sclerosis. These studies have shown that the extent of demyelination and remyelination is the same in new and chronic lesions and that remyelination is incomplete in most lesions.^142^ This also suggests that completely demyelinated lesions, which are common in histopathology, represent lesions that must have undergone multiple episodes of demyelination and incomplete remyelination. While longitudinal studies on MWF in multiple sclerosis lesions are rare, they also highlight the dynamic changes of lesions, with only 11% of silent lesions showing no change over a period of 2 years.^143^ Inhomogeneous MTR is believed to be particularly sensitive to highly restricted protons in lipid chains, making it more specific to the phospholipid bilayer of myelin compared to other MT imaging methods and MWF.^144,145^ Inhomogeneous MTR has been found to be reduced in WM lesions and normal-appearing WM compared with control WM, and reduced in WM lesions compared with normal-appearing WM.^146,147^

Considering myelin changes in the ageing brain also raises the question of how ageing *per se* affects magnetic resonance measures of myelin content and integrity.^148^ The most relevant MRI feature that changes with age is an increase in water content that begins around age 50 and is associated with prolonged T_1_ and T_2_ relaxation times.^149^ While changes in relaxation times are not expected to impact quantitative myelin measurements, subtle loss of microstructure and increased perivascular space have been shown to be a significant cause of underestimation of MWF in the ageing brain.^150^ This may also be true for the MTR, but it is not yet proven.

## MRI to measure treatment effect in the ageing multiple sclerosis patient

MRI parameters, typically the presence of new/newly-enlarging T_2_-hyperintense and gadolinium (Gd)-enhancing WM lesions on follow-up scans, are central in the definition of treatment response scores in patients with multiple sclerosis.^151^ However, group-level treatment efficacy^6^ shows a decreasing trend with increasing age, probably due to less MRI-visible inflammation.^152^ Conversely, older patients tend to show incidental T_2_ WM hyperintensities, mostly of vasculo-ischaemic origin.^153^ Therefore, the question arises as to whether monitoring the appearance of new lesions in follow-up scans is the most appropriate way to assess treatment response in the ageing patient. Unfortunately, no studies have focused on the definition of treatment response in patients older than 55 years, but lessons can be learned from discontinuation studies mostly targeting older populations and from *post hoc* analyses of randomized controlled trials, as well as real-world studies looking at the specific impact of age on treatment effect on MRI inflammatory markers.

The recent treatment discontinuation DISCOMS trial^154^ included stable (no relapse or new MRI lesions in the previous 3 years) multiple sclerosis patients older than 55 years of age (for a median age of 62/63 years for both trial arms) who were randomized to discontinue or maintain their disease-modifying drug. New T_2_-hyperintense WM lesions were observed in 3.9% of patients treated (10.7% in discontinued patients) over the 24 months of the study; this figure is much lower than that observed in treatment response studies,^151,155^ which is ∼50%. Nonetheless, caution should be exercised as these figures are not directly comparable due to relevant design differences. Of note, in the DISCOMS trial, the presence of comorbidities did not increase the risk of new T_2_-hyperintense WM lesions, indicating that the use of specific markers to detect new multiple sclerosis lesions (i.e. PRLs or lesions with the CVS) may not be needed. A *post hoc* analysis of the natalizumab trials^156^ looking at the impact of age on treatment effect has also shown that older age is associated with a lower prevalence and degree of focal inflammatory activity in both the placebo and in the interferon and natalizumab-treated arms. Unfortunately, no patients older than 55 years were included in these trials, but such a trend may likely be maintained beyond that age. Again, a recent real-world study cohort including 30% of patients beyond 40 years of age found that older age was associated with lower risk of MRI activity over follow-up in treated patients.^157^

In summary, even though age is associated with a lower risk of MRI-measured inflammatory activity, a higher risk of disease progression is observed with increasing age in multiple sclerosis patients. Such trends are also observed in treated patients, thus monitoring active inflammation to assess treatment efficacy and effectiveness does not seem to be advisable. Other MRI parameters (e.g. brain volume changes and slowly expanding lesions) should be studied in this age group to make sure that the pathological underpinnings of treatment response are adequately gauged.

## Conclusions

Peculiar immunological and pathological changes as well as a higher prevalence of comorbidities occurs with ageing. These factors may have substantial detrimental effects on disease evolution in addition to multiple sclerosis-related pathology in older patients. As the prevalence of ageing multiple sclerosis patients is constantly increasing, it is fundamental to investigate the clinical, immunopathological and MRI features of ageing in multiple sclerosis. The application of different MRI techniques that are sensitive and specific to the different pathological processes of multiple sclerosis may offer a substantial and clinically relevant contribution to allow a timely and accurate diagnosis in this peculiar population, limiting the risk of misdiagnosis, as well as to optimize monitoring of treatment to improve the clinical evolution of ageing multiple sclerosis patients. A deeper understanding of the evolving dynamic pathophysiological processes that may be peculiar of an older age may also contribute to the identification of new potential targets for future neuroprotective therapeutic strategies.

## Supplementary Material

awae251_Supplementary_Data

## Appendix 1

Collaborators: Ludwig Kappos, Gabriele De Luca, Menno Schoonheim.

The authors and collaborators are members of the MAGNIMS Study Group (www.magnims.eu), a group of European clinicians and scientists with an interest in undertaking collaborative studies using MRI methods in multiple sclerosis. The network is independent of any other organization and by the time the workshop this work is based upon was run by a steering committee whose members were: M. A. Rocca, J. Sastre-Garriga, F. Barkhof, O. Ciccarelli, N. de Stefano, M. Filippi, Claudio Gasperini, L. Kappos, Gabriele De Luca, C. Enzinger, À. Rovira, M. Schoonheim, and T. Yousry.
