# Supplementary material for: The ageing central nervous system in multiple sclerosis: the imaging perspective
Source: Brain. 2024 Jul 24;147(11):3665–80. doi: 10.1093/brain/awae251 (PMC11531849; doi:10.1093/brain/awae251)
Supplement: awae251_Supplementary_Data [file awae251_supplementary_data.pdf]

Attendees of the MAGNIMS workshop: "Imaging the aging CNS in multiple sclerosis" (Milan, November 10th, 2023)

Chairs - Massimo Filippi (Neuroimaging Research Unit, Division of Neuroscience, Neurology Unit, Neurorehabilitation Unit and Neurophysiology Service, IRCCS San Raffaele Scientific Institute, Milan, Italy; Vita-Salute San Raffaele University, Milan, Italy); Maria A. Rocca (Neuroimaging Research Unit, Division of Neuroscience, Neurology Unit, IRCCS San Raffaele Scientific Institute, Milan, Italy; Vita-Salute San Raffaele University, Milan, Italy)

Speakers – Andrea Cossarizza (Modena, Italy), Ruth Geraldes (Oxford), Cristina Granziera (Basel), Lukas Haider (Vienna), Hans Lassmann (Vienna), Giuseppe Pontillo (London), Paolo Preziosa (Milan), Stefan Ropele (Graz), Jaume Sastre-Garriga (Barcelona).

Discussants – Frederik Barkhof, Olga Ciccarelli, Nicola De Stefano, Claudio Gasperini, Monica Margoni, Alex Rovira, Tarek Yousry.

Supplementary Table 1. Search strategy and selection criteria

**Sources**	Pubmed (https://www.ncbi.nlm.nih.gov/pubmed)
**Period of time covered**	From January 1979 until April 2024
**Search terms**	References for this Review were identified through searches of PubMed (https://www.ncbi.nlm.nih.gov/pubmed) with the search terms "Aging", "Atrophy", "Brain aging", "Central vein sign", "Cerebral small vessel disease", "Chronic active lesions", "Comorbidities", "Cortical lesions", "Diagnostic Criteria", "Differential Diagnosis", "Glymphatic system", "Gray matter", "Immunosenescence", "Inflammageing", "Inflammation", "Iron", "Late-onset", "Lesion/s", "Magnetic resonance imaging", "McDonald criteria", "Multiple sclerosis", "Myelin", "Paramagnetic rim lesions", "Pathology", "Primary progressive", "Progressive", "Quantitative Susceptibility Mapping", "Secondary progressive", "Senescence", "Slowly expanding lesions", "Spinal cord", "Susceptibility-weighted imaging", "Treatment", "Virchow-Robin space", "White matter".<br>Only papers published in English were reviewed. The final reference list was generated with the consensus of all co-Authors of this review on the basis of originality and relevance to the broad scope of this Review, with a focus on articles published during the past five years.
**Selection criteria and review preparation**	1. Only papers published in English.<br>2. The final reference list was generated with the consensus of all co-authors of this review on the basis of originality and relevance to the broad scope of this review, with a focus on the most recent articles published in the last five years.<br>3. Experts provided a summary during the meeting of the main findings related to specific topics of the review. For each topic, a group consensus was reached and summarized in a first draft, which was circulated among the co-authors for further critical

	discussion and revision. The review represents the final conclusions reached by co-authors.
